# SELID: Selective Event Labeling for Intrusion Detection Datasets

**DOI:** 10.3390/s23136105

**Published:** 2023-07-02

**Authors:** Woohyuk Jang, Hyunmin Kim, Hyungbin Seo, Minsong Kim, Myungkeun Yoon

**Affiliations:** Department of Computer Science, Kookmin University, 77, Jeongneung-ro, Seongbuk-gu, Seoul 02707, Republic of Korea; spectat@kookmin.ac.kr (W.J.); clw3wb@kookmin.ac.kr (H.K.); antifly55@kookmin.ac.kr (H.S.); alsthd14@kookmin.ac.kr (M.K.)

**Keywords:** security operations center, intrusion detection, unsupervised learning, alert fatigue, cyber security

## Abstract

A large volume of security events, generally collected by distributed monitoring sensors, overwhelms human analysts at security operations centers and raises an alert fatigue problem. Machine learning is expected to mitigate this problem by automatically distinguishing between true alerts, or attacks, and falsely reported ones. Machine learning models should first be trained on datasets having correct labels, but the labeling process itself requires considerable human resources. In this paper, we present a new selective sampling scheme for efficient data labeling via unsupervised clustering. The new scheme transforms the byte sequence of an event into a fixed-size vector through content-defined chunking and feature hashing. Then, a clustering algorithm is applied to the vectors, and only a few samples from each cluster are selected for manual labeling. The experimental results demonstrate that the new scheme can select only 2% of the data for labeling without degrading the F1-score of the machine learning model. Two datasets, a private dataset from a real security operations center and a public dataset from the Internet for experimental reproducibility, are used.

## 1. Introduction

The security operations center (SOC) has a history that dates back more than 20 years, and it plays a key role in cyber security, especially in the attack detection and response [1,2,3]. When an intrusion detection system (IDS) is deployed as a security monitoring sensor, events or alerts are triggered by suspicious packets or abnormal flows. These events are generally short messages that are collected by a central server, called the security information and event management (SIEM) or security orchestration, automation, and response (SOAR) for further analysis. In this paper, we focus on a dataset of events collected by distributed monitoring IDS sensors. A modern IDS can block suspicious network packets after detecting any intrusion, and this is known as an intrusion prevention system (IPS). In this paper, we focus on detection, and “IDS” and “IPS” are used interchangeably.

There is a problem in that the number of events generated by these monitoring sensors is too large, especially the number of false-positive events (FP) that are falsely triggered, and this causes an alert fatigue problem [2,3,4,5,6,7]. An alert fatigue problem is the phenomenon in which many false-positive events occur, such that security officers tire of analyzing false-positive events and do not analyze actual attack events, or true-positive events (TP) [8,9]. According to a 2019 report by Cisco, 41% of the 3540 organizations they surveyed reported that more than 10,000 attack detection events occur every day, and only 24.1% of the analyzed events are actual attacks [8]. Although mitigating the alert fatigue problem is extremely important in practice, it has not been studied sufficiently due to the difficulties of dataset sharing. Recently, however, some studies on the alert fatigue problem have begun to be published [2,3].

Recently, machine learning models are expected to mitigate the alert fatigue problem by automatically distinguishing true-positive events from false-positive events. However, in order to develop a machine learning model that can achieve an excellent performance, datasets with labels of good quality should first be obtained. Unfortunately, the labeling process again requires considerable human resources, because a large volume of events are triggered by distributed IDS sensors. Therefore, we need to first select a manageable number of events from the entire dataset, and then only the selected events are manually labeled because of scarce human resources. After a machine learning model is trained on the labeled data, the model will automatically distinguish true positives from false positives.

In this paper, we present a new sampling and labeling scheme, called selective event labeling for intrusion detection datasets (SELID), which is based on unsupervised clustering. In the new scheme, a small number of events from a dataset with no labels are selected, and only the selected events are labeled by human analysts. Then, a supervised machine learning model is trained on the labeled events. Ideally, the performance of the supervised model using SELID should be almost the same as that of a model trained on a whole dataset that has been fully labeled by analysts.

Before SELID selects events, each event is converted into a vector through content-defined chunking (CDC) [10], feature hashing [11], and an algorithm of high-speed prototype clustering [12,13]. In this paper, we assume that a dataset consists of network-based IDS events, but SELID can be applied to any dataset of different event types. The contributions of this paper are summarized as follows:

- SELID can automatically select a small number of events from the dataset for the purpose of efficient data labeling. We present a content-based vectorization and clustering scheme that can make groups of similar events.
- We verify the effectiveness of SELID with one private dataset from a real security operations center and one public dataset from the Internet for experimental reproducibility.
- The experimental results demonstrate that SELID can reduce the number of labeling events by orders of magnitude without degrading the performance of the machine learning model.

We focus on efficiently selecting events from a given dataset that are manually labeled. Although the final goal of this would be to train a machine learning model with high accuracy, especially in supervised learning, developing a machine learning model is beyond the scope of this paper. We present SELID to select event samples without significantly degrading the model’s performance.

The rest of the paper is organized as follows. Section 2 presents the related work. We present SELID in Section 3 and the experimental results and discussion in Section 4. Section 5 presents the conclusion.

## 2. Related Work

The SOC has played a crucial role in cyber-security for 20 years, but it has only recently begun to be studied academically [1,2,3,14]. For example, Ede et al. presented a research paper where deep learning techniques were presented to address the issue of alert fatigue [3]. The paper proposed a method for mitigating the alert fatigue problem in SOC using unsupervised deep learning. The sequential order of events is memorized by the deep learning model, and similar event sequences observed in the past are processed in the same way, which reduces the number of events that security analysts must cope with. It is expected that more research will be conducted through industry–academia cooperation in the future.

Security devices, including IDS and IPS, are developed to capture various types of attacks to maintain a competitive edge in the market. In particular, it is a challenging task to define a precise and detailed rule for attack detection in IPS that can prevent false positives [15,16]. Additionally, as new attacks continue to appear along with the new rules, security devices generate a greater number of false-positive events, which leads to the serious issue of alert fatigue. This causes security analysts to experience burnout, and really important attacks may go unnoticed [2,3,4,5,6,7,8,9,17].

Hassan et al. proposed a method for prioritizing abnormal server logs and events using graph theory [7]. The advantage of a graph-based analysis is that it makes the causes and effects of abnormal events easier to find.

Shen et al. proposed a method to learn an event sequence using a recurrent neural network (RNN) as a deep learning method and to detect anomalies that were not observed during the training phase [18,19]. They focused on building an RNN model using event data from host-based IPSs [18]. The accuracy was approximately 90%, and they conducted experiments by collecting 3.4 billion detection events over four months from 740,000 Symantec IPS devices. The same research group also introduced ATTACK2VEC [19], which treats IPS events as sentences in natural language processing. Each event was embedded as a unique vector, which made it possible to circulate the similarity between the embedded event vectors using cosine similarity.

## 3. SELID: Selective Event Labeling for Intrusion Detection Datasets

**Figure 1:**
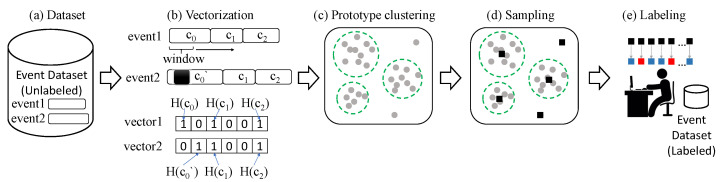
Processing steps for SELID.

SELID takes as input a large volume of IDS event data that have not been labeled at all and selects a small number of events for labeling. We assume that only *n* events from the dataset can be labeled by human analysts because human resources are scarce. SELID should select *n* events from the dataset so that the labeled events can lead to a machine learning model of high accuracy. Figure 1 shows the overall processing steps for SELID, each of which is explained in detail.

### 3.1. Dataset

We assume that a dataset consists of IDS events generated by distributed IDS sensors that monitor network packets and trigger events for packets that include attack signatures. We observe that recent IDS sensors attach a suspicious packet to a triggered event, which definitely gives helpful information to security analysts [20,21]. Because a packet is generally less than 1500 bytes in length, the size of the event is not excessively large. For an event reported to security analysts, they label it as true positive if the event is really related to cyberattacks; otherwise, they label it as false positive. We assume that an event is labeled either a true positive or false positive since the event has already been caught by IDS.

### 3.2. Vectorization

An event is a short byte sequence; for example, a packet payload is less than 1500 bytes in length. We transform an event into a fixed-size vector based on the contents of the payload, as shown in Figure 1b.

The packet payload of an event can be either a text or a binary; a text can be parsed by its white spaces or special characters. However, a binary such as an image or compressed contents cannot be parsed. In this paper, we adopt a content-defined chunking (CDC) algorithm to divide both text and binary payloads into multiple non-overlapping pieces, called chunks [10]. The Rabin fingerprint, or a rolling hash function, has commonly been used as a CDC algorithm in the past [22]. In this paper, we use the AE chunking algorithm, which is faster and more efficient than previous rolling hashes [10]. The AE algorithm divides the packet payload of an event into multiple chunks based on the payload contents or byte sequences, and we use the set of chunks in the next step. The window size of AE is set to four in this paper, which means that the average length of the chunks becomes (e−1)×4=6.87 bytes [10]. Similar events are transformed into similar sets. For example, event1 and event2 are the same except for one byte, colored as black in Figure 1b; their representative sets $\left\{c_{0},c_{1},c_{2}\right\}$ and $\left\{c_{0}\prime ,c_{1},c_{2}\right\}$ are also similar to each other, where only one element is different.

The representative set of an event is again transformed into a fixed-size representative vector [11]. In this paper, the default size of a vector is set to 512 and initialized to all zeros. We use feature hashing, also known as the hashing trick, to generate a vector. A hash function is applied to each element of the representative set, and the hashed value becomes an index over the vector, as shown in Figure 1b. The indexed value of the vector is then increased by one. We use feature hashing because it is a fast and efficient scheme for transforming any set into any fixed-size vector.

### 3.3. Clustering

After the events are transformed into vectors, we apply a clustering algorithm to generate clusters. Because a vector already reflects the contents of the original event, the clustering is based on the contents of the events. As we prefer a fast and lightweight clustering algorithm, we implement the prototype clustering proposed in [12,13], instead of well-known algorithms such as K-means and DBSCAN from open source libraries [23].

**Figure 2:**
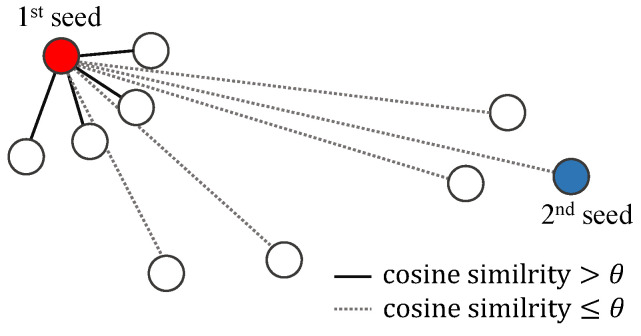
Prototype clustering.

In prototype clustering, a seed vector is chosen first, and then all other vectors that are similar to the seed by more than θ are considered as belonging to the same cluster of the seed vector. We set θ to 0.8 in this paper to cluster conservatively. The similarity, or distance, is measured by the cosine similarity. When a cluster is generated, the next seed vector is chosen by selecting the vector that is least similar to the previous seed vector, as shown in Figure 2. Then, all other vectors that do not yet belong to any cluster are checked to determine whether they are similar to the current seed vector in terms of θ. This process iterates until all vectors belong to a cluster.

**Figure 3:**
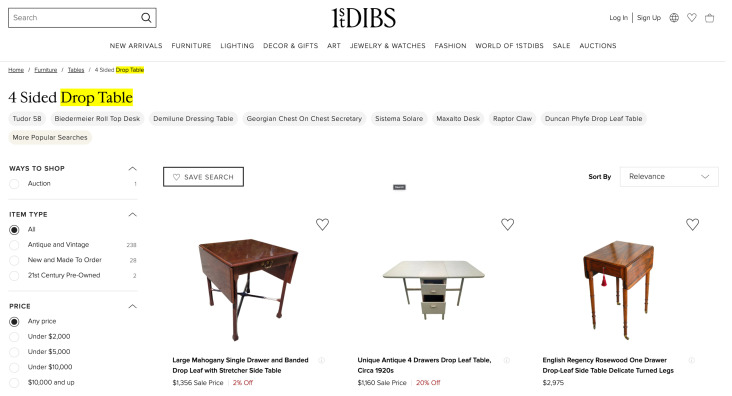
Example of a false-positive event by SQL-Injection [3].

We explain why content-based vectorization and clustering from an event dataset is useful for SELID. We observe that an event dataset often includes a large volume of events with similar contents, which is especially true for false-positive events, because every monitoring site has its own triggers of false-positive events; for example, Figure 3 shows a commercial web site that includes a product search phrase “drop table”, which is a piece of furniture. However, this phrase is also an IDS detection signature for detecting and preventing an SQL injection attack [3]. If a “drop table” appears for every customer connection, this would trigger a large volume of false positives. The packet payloads may look a little different for different sessions, as packet meta information such as time and length can change. Using SELID generates a cluster that includes all of these false positives, which saves a significant amount of human resources for the labeling task.

### 3.4. Sampling and Labeling

After the clustering is finished, SELID extracts samples from each cluster. We assume that only *n* events can be annotated by analysts due limited human resources and costs. Therefore, the maximum number of events in total should be *n*. The number of clusters is denoted as *r*. In this paper, we randomly select n/r events from each cluster, as shown in Figure 1d. We confirm that this simple sampling strategy works well in experiments.

Finally, security analysts label only the samples selected from the clusters, as shown in Figure 1e. The total number of labeled samples is *n*. The labeling time and quality depend on the skill and experience of the human analysts; according to a recent survey, it takes more than 20 min, on average, to process one event [7]. Even if one person works for 24 h without a break, only 76 events can be analyzed and labeled. Therefore, SELID is definitely necessary for any SOC.

## 4. Experiments and Discussion

We evaluate SELID in two datasets: one private dataset from a real security operations center and one public dataset from the Internet for experimental reproducibility. The experimental results demonstrate that SELID can select only 2% of the original event dataset for the purpose of data labeling without degrading the accuracy-related metrics of the machine learning model. We believe that SELID can reduce the labeling overhead of SOCs.

### 4.1. Experimental Setup

The SELID experiments are conducted on a desktop PC consisting of Intel(R) Core(TM) i9-9900X CPU @ 3.50 GHz, 128 GB memory, and TITAN RTX 4-way GPU, and the software includes open libraries such as Python, PyTorch, and scikit-learn.

The components of SELID shown in Figure 1 are all implemented in Python, including vectorization, prototype clustering, sampling, and labeling. Although the purpose of SELID is to select only a small number of events for labeling from a large volume of data, we must confirm that the performance of the machine learning model does not degrade. Therefore, we also implement a simple deep-learning model that is trained twice; first, the model is trained with all of the dataset with its original labels. We call this model a RAW model, or RAW for short. Second, the model is trained on the same dataset, but with the labels annotated by SELID; only *n* events are chosen from the SELID clusters and labeled. We call this the SELID model, or SELID for short.

**Figure 4:**
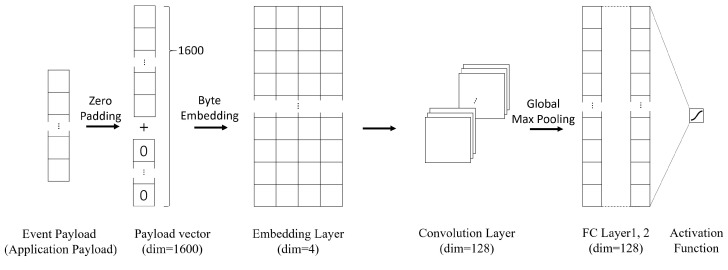
Supervised CNN model for SELID experiments.

Figure 4 shows the supervised deep-learning model that is used in this paper. The model is based on the convolutional neural network (CNN) proposed in [24,25]. The model divides the packet contents into byte-level features and passes them through an embedding layer, a convolution layer, and finally a fully-connected layer. The model checks the correlation between each byte in the packet to determine whether the event is either false positive or false negative. We assume that the maximum length of the application payload is 1500 bytes; if the length is less than 1500 bytes, zero padding is added. If the length is larger than 1500 bytes, we truncate the last bytes. We use the binary cross entropy as a loss function, and we adopt the ADAM optimizer. We also use ReLU as a non-linear activation function, and we use the sigmoid function in the last step.

The purpose of this experiment is to demonstrate the usefulness of SELID rather than to optimize the classification performance of the CNN model. Therefore, we use the same CNN base model with the same hyperparameter values for both the RAW and the SELID models. The number of training epochs for the private dataset is set to 32, and the training epochs for the public dataset is set to 64. The batch size is set to 128 for both datasets.

### 4.2. Experimental Datasets

Two different datasets are used for the experiments. The first one is from a real SOC and is used to verify the practical use of SELID. The second one is from the Internet and is used for experimental reproducibility [26]. We call them the private dataset and the public dataset, respectively.

In this paper, we divide each dataset into two parts of train and test to measure how SELID affects the performance of a machine learning model. Therefore, SELID is used on the train part. The same model, which is shown in Figure 4, is trained twice, once on the original training dataset and once on the SELID dataset. The results are compared in terms of accuracy, precision, recall, and F1-score, which are defined as follows:

- Accuracy = $\frac{TP\;+\;TN}{TP\;+\;TN\;+\;FN\;+\;FP}$;
- Precision = $\frac{TP}{TP\;+\;FP}$;
- Recall = $\frac{TP}{TP\;+\;FN}$;
- F1-score = $2\times \frac{Precision\;\times \;Recall}{Precision\;+\;Recall}$.

Where TP, TN, FP, and FN are explained as follows:

- True Positive (TP): An attack event is classified as an attack event.
- True Negative (TN): A non-attack event is classified as a non-attack event.
- False Positive (FP): A non-attack event is classified as an attack event.
- False Negative (FN): An attack event is classified as a non-attack event.

We believe that the F1-score considers both precision and recall, and therefore this metric effectively demonstrates the overall performance of the model.

#### 4.2.1. Private Dataset

The first dataset was provided by one of the largest SOCs in South Korea for academic purpose only. The SOC has 10 different IDS monitoring sensors deployed at different sites. Each sensor is located at the Internet gateway and inspects network packets to detect cyberattacks on the site. The attack types include cross-site scripting (XSS), SQL injection, malware download, distributed denial of service (DDoS), connection from blacklist IP addresses, etc. The dataset has 1,276,275 events in total, and all events have already been labeled as either true-positive or false-positive events by the SOC. Table 1 shows the statistics for the private dataset.

#### 4.2.2. Public Dataset

The second dataset is an open dataset of web traffic, which is published on the Internet and used in many papers [26]. The dataset is composed of web request packets, and each packet is labeled as either normal or anomalous. In this paper, normal labels are considered as false positives while anomalous labels are true positives. The dataset was originally designed to detect abnormal packets. In this paper, we use the dataset to demonstrate the performance of SELID for experimental reproducibility. Table 2 shows the statistics for the public dataset.

### 4.3. Experimental Results

We demonstrate the experimental results. First, the experimental results of the private dataset are presented, followed by the results for the public dataset. The experimental results are presented in terms of both the reduction in labeling efforts and the maintenance of accuracy.

**Figure 5:**
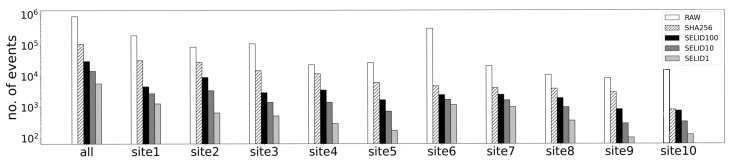
Comparison of the number of events selected by SELID for different sites. SELID10 selected only 1.71% of the original dataset events when the private dataset is used.

**Figure 6:**
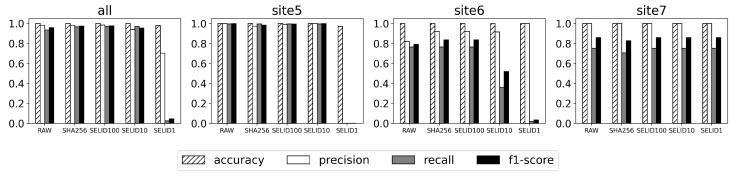
Performance comparison of deep learning models on SELID-selected dataset vs. original dataset when the private dataset is used.

**Private dataset.**Figure 5 and Figure 6 demonstrate the experimental results of SELID. The numbers of events selected by SELID for different sites is compared in Figure 5; RAW is the number of events in the original dataset. We duplicated events of the same contents by SHA256 fingerprinting, and the duplicated dataset is denoted as SHA256. We implement three different versions of SELID; SELID1 randomly selects one sample per cluster, SELID10 selects 10 samples per cluster, and SELID100 selects 100 samples per cluster. We compare the reduction effect and the model performance against the different numbers of samples selected by SELID.

As shown in Figure 5, SELID1, SELID10, and SELID100 selected only 0.66%, 1.71%, and 3.50%, respectively, of the events from the original dataset. Figure 6 shows that the machine learning model trained on the SELID1 dataset degrades in terms of precision, recall, and F1-score. Comparing these figures reveals that SELID100 maintains the performance of the machine learning model almost as well as RAW. Except for site6, SELID10 maintains the performance almost as well as RAW. This means that sampling only 1.71% of events was sufficient for training the supervised model, and human efforts for the manual analysis of 98.29% events could be saved.

**Public dataset.** The same experiment was performed on the public dataset of the web request traffic [26]. Again, we first measure the number of events that are selected by SELID. Then, we compare the performance of the machine learning models that are trained on the different numbers of events selected by SELID.

**Figure 7:**
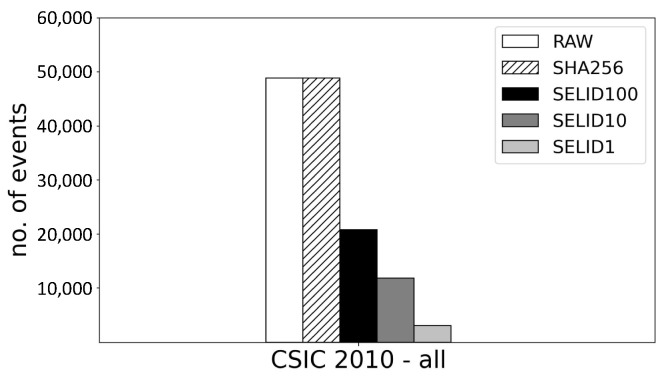
Comparison of the number of events between different sites when the public dataset is used.

Figure 7 compares the number of event samples selected by SELID, the original number of events in total (RAW), and the number of events with content-based, duplicated (SHA256). It is interesting that RAW is equal to SHA256, which implies that there are no packets of the same content. This figure reveals that SELID1, SELID10, and SELID100 selected only 6.75%, 17.97%, and 34.73% of events compared with RAW, respectively. Because this dataset is far less realistic than the private dataset, a relatively large number of events are selected by SELID.

**Figure 8:**
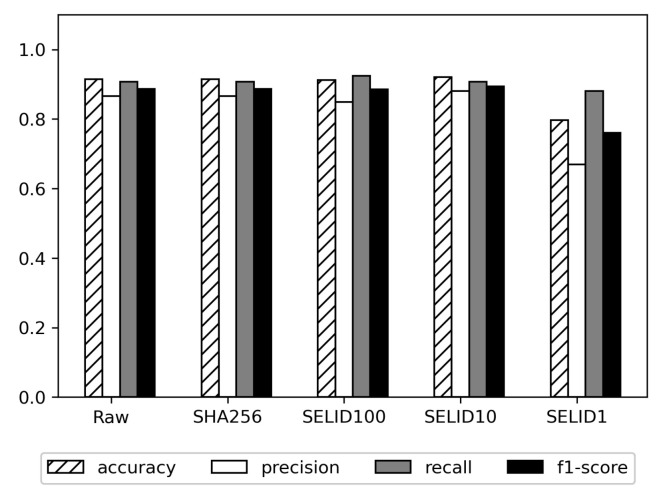
Performance comparison of deep learning models on SELID-selected dataset vs. original dataset when the public dataset is used.

Machine learning models trained on different data are compared in Figure 8. We confirm that SELID again maintains the performance of the model as well as RAW, except for the SELID1.

### 4.4. Discussion

From the experimental results, we confirm that SELID maintains the performance of a machine learning model while selecting only a small number of events for labeling. We believe that security events are redundant because false positives are triggered by similar packets, as shown in Figure 3, and even true positives are triggered by the same attack tools, which generate similar packets. Therefore, once clusters are generated on packet contents, SELID can select a few samples per cluster as good representative events.

We also confirm that the reduction ratio of the private dataset is much better than that of the public dataset. For example, only 1.71% of events in the private dataset were selected by SELID10, whereas 17.97% of events in the public dataset were required for SELID10. A real dataset would include a greater number of redundant elements because the human resources of an SOC are too expensive to be used to check and reduce strong redundancy among data. However, the open dataset could be checked many times by its creators. For example, the SHA256 is equal to RAW on the public dataset. We believe that SELID could help any real SOCs by automatically selecting only a small percentage of samples from an original dataset.

We believe that SELID can be applied to any datasets where similar contents imply similar attack types and labels. For example, malware files from the same attacker may be similar to each other in terms of certain features such as a byte sequence, API call sequence, and printable strings [27]. In this case, SELID can be used to cluster similar malware files into the same group to save human resources to analyze the whole malware files. We leave the application of SELID to other datasets in future work.

## 5. Conclusions

In this paper, we propose SELID, which selects a small number of security events for labeling while maintaining the performance of a machine learning model. This saves a significant amount of human efforts and time because data labeling at an SOC is a time-consuming task, and the sheer volume of event data has made it impossible to manually select representative samples until now. We believe that SELID can significantly reduce the event labeling workload at SOC.

## Figures and Tables

**Table 1:** Statistics for the private dataset.

Site	Train		Test	
	FP	TP	FP	TP
1	346,002	2016	54,448	119
2	202,835	4135	190,130	6187
3	10,564	541	6889	403
4	26,883	489	12,792	384
5	22,884	563	84,238	1397
6	87,903	24	10,554	24
7	6599	1952	1295	160
8	112,939	117	4415	21
9	17,050	2	21,263	16
10	21,484	43	16,447	68
all	855,143	9882	402,471	8779

**Table 2:** Statistics for the public dataset [26].

	Train	Test
FP	28,800	20,052
TP	7200	5013

## Data Availability

The dataset includes security-related information and therefore should not be open to public. Please contact the corresponding author for the dataset if you need it.
